# Normative preconditions for the assessment of mental disorder

**DOI:** 10.3389/fpsyg.2013.00611

**Published:** 2013-09-09

**Authors:** Marco Stier

**Affiliations:** Institute for Medical Ethics, History and Philosophy of Medicine, University of MuensterMuenster, Germany

**Keywords:** biological psychiatry, norms, reduction, cultural relativity, morality

## Abstract

The debate about the relevance of values for the concept of a mental disorder has quite a long history. In the light of newer insights into neuroscience and molecular biology it is necessary to re-evaluate this issue. Since the medical model in previous decades was more of a confession rather than evidence based, one could assume that it is—due to scientific progress—currently becoming the one and only bedrock of psychiatry. This article argues that this would be a misapprehension of the normative constitution of the assessment of human behavior. The claim made here is twofold: First, whether something is a mental disease can only be determined on the mental level. This is so because we can only call behavior deviant by comparing it to non-deviant behavior, i.e., by using norms regarding *behavior*. Second, from this it follows that psychiatric disorders cannot be completely reduced to the physical level even if mental processes and states as such might be completely reducible to brain functions.

## Introduction

In the course of the “molecular turn” (Rudnick, 2002) in psychiatry, researchers purport to “provide more objective diagnoses” (Akil et al., 2010, p. 1581) with the help of biological markers. Our traditional diagnoses, they claim, are not only unhelpful but actually a handicap for causal research (Holsboer, 2010, p. 1308). This is why “psychiatric disorders should be reclassified as disorders of the (central) nervous system” (White et al., 2012, p. 1). Even the neurosciences seem to have lost their leading position and appear to have gotten diminished to merely heuristic value since the “real” discoveries are to be expected on the molecular level (Bickle, 2006). While the adherents of the disease (or medical) model of mental^1^ disorder purport that psychiatry is at least as value free as all the other sciences, critics claim that psychiatry rests on norms and values *over and above* those being present in, say, physics or chemistry, since it deals with the mental, i.e., the experiences, emotions, and behaviors of persons, and therefore always includes norms in respect to these phenomena.

It would be trivial claiming that even the criteria for something being a brain defect rest on norms and that, hence, the criteria for a mental disorder cannot be norm-independent either because they rest upon brain defects. The claim made here is twofold: First, whether something is a mental disorder can only be determined on the mental level. This is so because we can only call a behavior deviant by comparing it to non-deviant behavior, i.e., by using norms regarding *behavior*, which simply are not applicable to neurons. The brain alone cannot give us the evidence necessary^2^. Second, from this it follows that psychiatric disorders cannot be completely reduced to the physical level, may it be neuronal or molecular. The classification of something as a mental disorder cannot even in principle be free of values and norms and can be “objective” only insofar as norms and values can be seen as objective. This is the case even if mental processes and states might—in principle as well—be completely reducible to brain functions. Hence, for the sake of the argument I will take the latter for granted: there is no behavior or experience, I assume, that does not come from the brain, and there is nothing in the mental realm that could not be reduced to the brain's processes. Nonetheless, whether a certain kind of behavior or experience should be seen as disordered, is *not* reducible to the brain's functions.

Thomas Szasz once stated: “It is not by accident that, in all the psychiatric literature, there is not a single account of voices that command a schizophrenic to be especially kind to his wife” and he continued, “[t]his is because being kind to one's wife is not the sort of behavior to which we want to assign a causal (psychiatric) explanation” (Szasz, 2001, p. 300). Even if we are not devoted adherents of Szasz, this quote should give us pause. There seems to be something peculiar about behavior that is beyond purely physical explanation because the difference between, say, acting kindly and unkindly can hardly be grasped in physical, non-normative terms.

In this paper I neither intend to offer another definition of mental disorder nor do I claim an incompleteness of some sort of neuroscience. Above all, I want to stress at the very beginning that I do not doubt the existence of mental disorders. If you have ever seen a deeply depressed person, or a schizophrenic desperately asserting his responsibility for the destruction of the WTC twin towers, you will not have any doubt about the existence of mental disorders. All I want to show is that mental disorders cannot be determined in a purely physical way.

In the following section I will explain my claim that psychiatric diseases are irreducible to the brain even if the mental as such may in principle be reducible. In the main part of the paper I will first show that psychiatry is embedded in several *normative frames of reference*, and then refer to five particularly relevant normative dimensions of psychiatry. These are the concept of *rationality*, *moral* assumptions, the notions of *harm and distress*, several *cultural* norms and influences, and finally the relevance of—equally normative—*routes of explanation*.

## The physical foundation of the mental

There is no behavior that does not arise from the brain. Neither is there something like a Cartesian soul, nor is there full-fledged mental causation. How can one nonetheless regard mental disorders as irreducible to neurobiology? Doesn't this look like wanting to have one's cake and eat it too? It might, at first glance, but things are not that simple.

If biological psychiatry was nothing but an ideology, as some authors claim (Cohen, 1993; Berger, 2001; McLaren, 2010), one would just have to show the irreducibility on this level. But we do not need to make such a principled assumption.

Let's assume every single aspect of our mental and behavioral life could be explained in purely physical terms. In this case it could not only be shown *that* our brains, together with our genetic endowment, are responsible for the way we are, but also *how* this happens, and which mechanisms are involved in producing this or that kind of thought or behavior. Let's further suppose the neurosciences could even explain the so-called phenomenal qualities—the “what it is like” to see red or to be depressed. Since what we call “mental disorder” is without doubt part of people's mental and behavioral lives, it would be explicable in purely physical terms as well. So it seems. To give an example: It would be possible to explain which of the brain's functions and properties make a person feel “depressed.” To make the claim even stronger, let's take for granted that environmental influences, too, are explicable mechanistically and that “[e]xploring the mechanisms of gene-environment interactions for depression is not substantially different from understanding how environmental toxins contribute to cancer or how diet influences cardiovascular diseases” as Thomas Insel and Remi Quirion assume (Insel and Quirion, 2005, p. 2221). Would we be able to determine what a mental disorder is by physical means alone? We wouldn't.

This is due to the fact that no behavior or inner feeling has a sticker on it that reads “I'm a disorder!” We have to write those stickers ourselves and attach them to certain feelings and behaviors. It is completely right when Matthew Broome and Paolo Fusar-Poli write:

“It is by observing how the person behaves with respect to her beliefs, and by witnessing such behavior in the process of the giving and asking of reasons that one suspects delusions, not in viewing a brain scan or a genetic sequence. In other words, the diagnosis of delusions is based on the observation of behavior that violates accepted norms (e.g., of rationality for belief reports).”

(Broome and Fusar-Poli, 2012, p. 598)

In short, whether something is a mental disorder has to be evaluated, not be discovered. This seems to be a purely Szaszian account, but it is not. According to Szasz, mental disorders are evaluated on a normative basis and not, as it is the case with physical diseases, discovered on the basis of functional or structural lesions. Psychiatric diagnoses “are driven by non-medical, that is, economic, personal, legal, political, or social considerations and incentives” (Szasz, 1994, p. 37). Up to this point I agree with Szasz. But while he claims that mental illnesses cannot be treated by medical means for this reason, I neither maintain this, nor do I dispute their existence. His argument seems to be something like this: (i) only medically discoverable conditions can be treated medically; (ii) mental illness is not medically discovered but normatively evaluated; (iii) mental illness cannot be treated medically. The argument fails because premise (i) is problematic. If we reformulate it into “only physically based conditions can be treated medically” the problem becomes obvious: Szasz confounds the epistemological and ontological side of the issue. All that can be inferred from the fact that mental illness is evaluated and not discovered is—at best—that there are no natural kinds of mental illness. We draw the line between normal and allegedly deviant behavior somewhat arbitrarily. But the question of how we can and should categorize forms (and norms) of behavior is different in kind from the further question of whether mental disorders exist. The first one is an epistemological question, the second one is ontological. Moreover, it is obvious that we can even “treat” completely normal behavior. Psychological enhancement gives the best evidence. This follows not at least from the assumption that no behavior or experience can exist without a brain producing it. Change the brain and you change the mind^3^.

While Szasz asserts mental illness does not exist because of its evaluative nature, my weaker claim is that it will never be possible to determine in a purely physical way which of the countless variants of behavior and thinking are disorders, even if we might discover all the physical causes of each and every thought and form of behavior one day. Hence, the irreducibility of mental disorders is not due to the mind-brain problem. But where exactly does the irreducibility come from? In the following section I will give an outline of the main normative aspects that prevent mental disorders from being explained purely physically.

## Normative bedrocks of mental disease

Stating that everything is normative insofar as we have to decide what kind of evidence we want to count as proof for something or what we are willing to accept as an explanation in science would be trivial. It would not be very shocking to claim that, e.g., neuroscientists have to use normative concepts such as the “correct functioning” of certain brain areas. Nearly everything in the world—including psychiatry—is normative in this sense. A much more provocative claim is that psychiatry is guided by social, moral, cultural and other norms. If this is true, and if it is also true that these kinds of norms are relative to time and place, then psychiatry cannot claim to know what a mental disease is “in itself,” where normality ends and mental disorder begins. Again, if the boundary between normality and mental disorder is a social construction such that the question of whether a certain kind of behavior is a disorder can only be judged against the background of this very convention, then the “disorderness” of a condition cannot be found on—and hence not be reduced to—the neuronal level. Psychiatry would have to admit that it serves—to a certain degree at least—not only the needs of patients but those of society as well.

### Normative frames of reference

Judgments of psychiatric disorder always need a background of psychiatric *order* without which no diagnoses could be made. A relatively easy way of finding such a background or “frame of reference” is to take a set of diagnostic criteria and turn them (back) into behavioral imperatives. Leising and colleagues have made visible the normative assumptions inherent in the DSM-IV criteria for personality disorders (PDs) in this way (Leising et al., 2009). To give just one example: On the basis of criterion one of Borderline and criteria seven and eight of Dependent PD they formulated the underlying norm “be able to tolerate real and imagined separation^4^.” If a person is not able to conform to this and other social standards she may be a candidate for a PD. It may be objected that this only refers to some single criteria while in the case of, e.g., Borderline PD seven out of nine criteria have to be met. This is true, of course. But what about the normativity of the other criteria? What do “unstable and intense interpersonal relationships” (DSM-IV-TR, 301.38, 2), or an “unstable self-image” (DSM-IV-TR, 301.38, 3) mean?

A principled objection against the normativity assumption could go like this: The current diagnostic manuals are indeed deeply misguided, but once we have found the *real* and *appropriate* criteria for psychiatric disorders, we will get rid of the normativity problem. But again, on the basis of what background or reference frame will such an ideal manual function? Since it is always experience and behavior that have to be judged as pathological, we will always have to draw on “average people” to tell apart mental and/or behavioral deviance on the one hand and “normality” on the other.

In particular, four such normative frames of reference can be distinguished (cf. Leising et al., 2009 for the following)^5^.

1. The personal values of a given diagnostician: In the absence of a strong theoretical foundation it is more likely than not that the criteria follow the values and worldview of those who establish them.
2. Cultural expectations: Diagnoses might not primarily refer to the person but to the mismatch between her patterns of culturally primed behavior and the expectations of her current social environment. For instance, western-style behavior of a girl in rural areas of Turkey may become a candidate for a PD. Conversely, rural Turkish behavior patterns may be seen as an indicator of a psychiatric disorder in the west.
3. Generalized assumptions about human nature: While it may be possible to determine something like “normal functioning” of the body, e.g., in respect to heart, liver, or the hormonal system, it is quite difficult, if not impossible, to find universal human mental and behavioral patterns. Even if there is a species-typical behavioral setup, it is questionable whether the thresholds to pathological behavior and thinking similarly follow species-typical patterns^6^.
4. *Harm and disturbance*: What constitutes harm for one person does not need to constitute harm for another. In particular, the thresholds to harm and the kinds of issues that are regarded as harmful differ from one culture to another. Therefore, harmfulness is always judged against the background of varying, contingent frameworks.

While these frames of reference are situated on a more general level, Sadler and Fulford have indicated seven normative judgments that are “nested” in the *individual* diagnostic act (Sadler and Fulford, 2006, p. 171 f.). These concern:

- a match of the criterion's semantic content against the patient's phenomenal clinical presentation;
- a judgment by the examiner about the appropriate approach to the solicitation of relevant data from a patient;
- an examiner judgment about the prevailing sociocultural norms relevant to a particular criterion;
- an appraisal of the patient's performance (behavior, interview discourse) relevant to said sociocultural norms;
- a comparison between the patient's performance and the specific sociocultural norms in determining whether the patient's performance substantively deviates from them;
- the determination of whether such deviance is substantive enough, qualitatively (e.g., idiosyncratic deviance, as in “bizarre delusions”) or quantitatively (e.g., as in “excessive” need for reassurance in dependent PD), to constitute psychopathology; and, finally,
- a judgment about whether the criterion-driven behavior and experience is disvalued or for the worse.

Apart from the respective diagnostic manual the diagnostician in a clinical setting cannot but make a whole range of normative judgments in individual cases. It is *in principle* impossible to get rid of this normative aspect of the task, even if the underlying biological mechanisms of a particular behavior or experience were completely known.

In the following I will discuss five normative dimensions that are present in psychiatry to varying degrees. The first is “rationality,” the role of which is somewhat underestimated in the discussion of the normative preconditions of psychiatry (section Rationality); the second refers to the special case of PDs which seem to be particularly dependent on moral expectations (section Morality); third, there is the problematic notion of “harm and distress” that has already been mentioned above (section Harm and Distress); fourth, we have to ask to what extend the concept of psychiatric disorder is relative to different cultural backgrounds (section Culture); the fifth normative dimension pertains to the relativity of scientific explanatory routes which are no less normative in character (section Routes of Explanation).

### Rationality

Even though “irrationality” and corresponding terms are not explicitly mentioned as criteria in the current versions of DSM or ICD, Marie Crowe has pointed out that there are several features to be found in the DSM with which a person's perception of reality must be consistent in order for the person to be attributed with rationality. These include notions such as “impairment in reality testing,” “magical thinking,” “suspects without sufficient basis, that others are exploiting, harming or deceiving him or her,” or “worry about everyday, routine life circumstances” (Crowe, 2000, p. 75). Yet, this does not say what kind of reality is at stake.

There are several concepts of rationality (Bunge, 2007), two^7^ of which are of particular interest in psychiatry: The first one is theoretical or linguistic in nature (logical rationality) while the second one is practical in the sense of means-end rationality (practical rationality). When someone concludes from (i) human beings are mortal, and (ii) Socrates is a human being, that (iii) Socrates is *im*mortal, his theoretical rationality has failed. If mental disorder could be characterized by a lack of theoretical rationality, things would be quite easy. Unfortunately, this is not the case. A couple of years ago a study was conducted showing schizophrenic people to be even more theoretically rational than average persons (Owen et al., 2007). Practical rationality, on the other hand, comes in degrees and is not always judged by the same standards. If a person who has become convinced by advertisement that a certain kind of caffeinated drink makes you popular and henceforth consumes it for this reason, we would probably attest a lack of practical rationality. If someone seeks a cure for cancer in prayer, this would be (at least in the eyes of many) a grave lack of practical rationality, too. Now think of a person who washes her hands every 10 min in order not to catch an infection. There are, of course, other forms of practical non-rationality which leave hardly any doubt that something must be wrong with a person. But we have to set the cut-off ourselves, and there is no other way than doing this somewhat arbitrarily.

The problem already begins with the assessment of capacity and competence to make treatment choices. While it could be argued that there is an objective way of assessing patients' capacity by testing their cognitive abilities to understand, retain and weigh up information, it is often overlooked that this is accompanied by a number of inherently normative judgments in clinical practice (Banner, 2012). Hence, it is not only the capacity of the patient that can be put into doubt, but also the way she makes use of it. And this aspect, the *way* of using information, cannot be assessed but on normative grounds. One of the most well-known examples in this regard is anorexia nervosa, where patients usually completely understand the relevant information and consequences but nevertheless make choices that other people would regard as problematic (see, e.g., Craigie, 2011).

The assessment of rationality in people's choices is normative in two respects. First, it is not always a precondition for recognizing the autonomy of a person; in some circumstances it is, in some it is not. Let's call this the “Switching-Standard-Thesis” (SST). Second, and connected to the first, the threshold beyond which a certain kind of irrational behavior can be seen as pathologic varies considerably. Call this the “Switching-Threshold-Thesis” (STT).

#### The switching-standard-thesis

According to SST the standard of rationality to which a person is expected to conform is the higher, the more she is suspected of having a psychiatric condition. As long as someone is regarded as “normal” her decisions may completely unreasonable in the eyes of others. As judge Lord Donaldson pointed out in an often quoted decision, the “right of choice is not limited to decisions which others might regard as sensible. It exists notwithstanding that the reasons for making the choice are rational, irrational, unknown or even non-existent” (Re “T.”, 1992). In a similar vein, Craig Edwards underscores that if someone ruins his reputation due to mental illness he may end up having to undergo involuntary psychiatric treatment, but if he does so without mental problems, it is his own business and he will not experience (strong) interventions (Edwards, 2009). While ordinary people are allowed to make irrational decisions even in highly important matters without being deemed incompetent (just think of decisions regarding the termination of treatment), patients with a suspected mental problem are at greater risk of being judged incompetent because of the very same “irrationality” (Banner, 2012). It is, therefore, a matter of normative choice and not one of objective judgment whether rationality is regarded as a component of mental health or not. It is usually being judged on normative grounds whether to examine someone's rationality further or not. If a mental disorder is suspected, we do; otherwise we don't. Irrationality is not the indicator of a mental problem. The dependency relationship runs the other way round: a suspected mental disorder is the reason why we take a closer look at someone's rationality and possibly regard a decision as irrational and incompetent that we otherwise would have accepted as competent.

#### The switching-threshold-thesis

Here it is not asked whether someone's rationality should be subjected to deeper scrutiny or not, but whether irrational behavior should be seen as indicating a mental problem. We all constantly behave irrationally in everyday life. It therefore has to be decided whether the irrationality of a person should count as part of a mental problem. Edwards lists a whole series of conditions such as greed, jealousy, hatred, or racial prejudice that impair our rationality and that “are sometimes considered to negative impact our well-being and that fall outside of our ability to control as rational agents, yet are not usually considered mental illnesses” (Edwards, 2009, p. 80). The threshold of rationality beyond which someone is being seen as having a psychiatric disorder is varying.

Both cases look very similar, and they indeed point to the same problem from different angles. According to SST, a mental disorder is diagnosed first, and subsequently a standard of rationality is applied that is higher than in everyday life. According to STT, irrational behavior that is judged to be normal on the background of one framework may be seen as indicating a mental disorder in other cases. The assessment of rationality is deeply normative.

### Morality

I should stress once more that my claim is not that *all* psychiatric disorders are moral in kind. What I do claim is, nevertheless, that many conditions—or conditions in many circumstances—at least *involve* (morally) normative elements and thus cannot be purely value free, non-normative (objective) medical kinds. The moral side of ascriptions of psychiatric disorders is most obvious in Cluster B PDs. Louis Charland uses two arguments to show this (Charland, 2006): The “*argument from identification*” and the “*argument from treatment*.” According to the first one, Cluster B disorders are identified in the DSM through *explicit moral* terms and notions such as “lying,” “lack of empathy,” or “conning others.” It would be hard to explain why a condition that is defined this way should not be moral in nature. His second point is only partly an argument on its own since it relies on the validity of the first one. What he has in mind seems to be that there is an important difference between, say, ceasing to be depressed on the one hand and ceasing to be a liar on the other. The difference is that the first case can be seen as a cure while the second case is “tantamount to a moral conversion” (Charland, 2006, p. 122).

Possible counterarguments to this account are not far to seek. First, one could argue that it is not the morally questionable behavior as such that defines the disorder but the respective person's inability to change it, her irresponsiveness to reasons. Even if this sounds comprehensible, on a closer look it becomes obvious that an immutability criterion like this one only makes sense in connection with a presupposed moral judgment. There is hardly any person in the world that can change her character traits from one moment or week to the other. Character traits which we would not even think of as pathologic can be as “hardwired” as a full-fledged “PD.” Think of a particularly polite and attentive man who has become this way through his genetic endowment and parental upbringing. Every morning he tells himself to be a bit more selfish—but he just can't help it. He cannot change his style of behavior, but hardly anybody would suspect a psychiatric problem here. Both character traits as well as dysfunctions cannot be overcome just by choosing to do so. Second, the availability of therapeutic help or treatment that could be seen as a distinguishing factor is not a good candidate criterion either. Edwards emphasizes this pointedly when he states that the “need for, or availability of, treatment does not make something an illness any more than plastic surgery makes a crooked nose an illness” (Edwards, 2009, p. 81). Third, neither are character and dysfunction discernible through underlying causes since wicked behavior is equally due to internal and external biological influences and environmental conditions as mental disorder is. With the appropriate chemicals (or even brainwashing methods) you can “treat” grandma's joy, little Johnny's nosiness, or Martha's politeness as effectively as Bill's full-fledged depression.

Edwards, who regards the concept of psychiatric disorder as morally based, realizes this very tension. His way out is a catalogue of five criteria, each of which is necessary but not sufficient, together with the assumption that there is genuine moral truth in the world. His criteria, formulated as questions, are the following: (a) Is the condition harmful for the person who has it? (b) Is there any reason for legitimizing the condition as a character trait that one can choose to develop or maintain? (c) Is the condition one that can be discouraged through the inculcation of appropriate moral values during childhood? (d) Will applying moral responsibility to the condition help to uphold broader moral values in one's ethical system? (e) Can one have insight into the condition's effect upon oneself and if so, how difficult is it to take an active role in seeking treatment for oneself? (Edwards, 2009, p. 83 f.) As one can see, all five questions can indeed help only if they have answers that are not themselves contestable and/or relative to society, culture, and underlying moral creeds. With his reference to ethical truths Edwards may at least avoid the lurking diagnostic arbitrariness, even if that makes psychiatric diagnostics no less moral. Those however, who do not belief in objective moral truths, are still lost in the wilderness of psychiatric relativity.

In a strictly religious society being an atheist may be seen as a dysfunction of personhood; when our western societies still were (regarded as) strictly heterosexual, homosexuality was regarded as dysfunctional and, hence, a mental disorder; since productivity is highly valued in our busy and buzzing western societies, lack of productivity has become a part of the definition of mental disorders (Crowe, 2000, p. 73).

### Harm and distress

One could assume that harm is not a normative concept: if a person suffers she suffers, period. In the context of psychiatric diagnosis things are more complicated, however. A first crucial point that illuminates the normativity of harm has been emphasized by Fulford (2002). We just don't realize the value-ladenness of physical harm because most people regard, say, a broken leg as something bad and painful. Values that are shared by most people tend to hide themselves behind their commonness. When it comes to mental suffering our values diverge to a certain degree. Hence, it is not that bodily diseases are value-free whereas psychiatric disorders are value-laden. Both rest on normative assumptions. In one field we simply share them, in the other we don't. As Fulford writes:

“Thus, the criteria for good and bad heart functioning, for example, paralleling ‘good strawberries,’ are largely settled and agreed upon, and this is true by and large of all the areas with which (acute) bodily medicine is primarily concerned. By contrast, however, the areas with which psychiatry is primarily concerned—emotion, desire, belief, motivation, sexuality and so forth—are all areas in which our values, paralleling ‘good pictures,’ are highly diverse.”

(Fulford, 2011, p. 3 f.)

The most prominent author to have included the concept of harm in his theory of disorder is probably Wakefield. According to his “*harmful dysfunction analysis*” (Wakefield, 1992) we first have a function of a certain mechanism that turns into a *dys*function if the mechanism does not properly perform the tasks it was designed for by evolution; and if this dysfunction is furthermore harmful for the respective person, then it becomes a disorder. It is therefore not enough to state a (physical or mental) mechanism's dysfunction, since there are lots of dysfunctions that are not seen as disorders^8^. On the other hand, we all experience many harmful things in life without regarding them as mental disorders. Harm, he rightly assumes, is a value concept because it is relative to cultural assumptions. While this is plausible, turning Wakefield's idea upside-down is plausible, too: It may well be that we first disvalue a condition as harmful and only then search—and find—a mechanism of some sort that has a dysfunction of some sort. This would only be impossible if we could have a look into God's (or the evolution's) model kit.

But there are even more normative aspects in the notion of harm. First, the harm criterion leaves open *who* has to judge whether a person feels harm and distress enough and whether it is pathologic in character. It is one thing to subjectively feel harm and distress, quite another is to judge whether distress is pathologic, and, if it is recognized as potentially pathologic, what degree someone's suffering must reach in order to warrant a psychiatric diagnosis. Second, particularly in the case of Cluster-B PDs it is often the social environment, i.e., other people, who experience harm due to the “patient's” condition while he himself feels fine. A successful, narcissistic person will probably feel no distress at all while the people around him may suffer considerably. Third, harm also can arise indirectly from one's acts and with a temporal delay. If someone in a manic phase makes highly risky and imprudent transactions, the “harm” will (a) be indirect because not the condition itself is harmful or distressing but its consequences may cause harm, (b) the harm caused may initially not represent a problem for the person in question but for his spouse or children, (c) whether a risky and imprudent financial transaction or its consequences should be seen as harmful is clearly nothing we can read off some diagnostic manual. Financial losses are to be judged economically, not medically. Even if the person later deeply regrets what she has done, it remains unclear what degree of regret will warrant a psychiatric diagnosis.

### Culture

One of the most widely discussed issues in the philosophy of psychiatry is the impact of cultural varieties on the concept of psychiatric disorder. Do different cultures give rise to special forms of disorder experience? Are there mental disorders that are due to particular socio-cultural frameworks? These and other questions have been disputed for a long time. There is one tradition that takes cultural particularities into account. It is called the “emic” approach. In contrast, the “etic” account tries to explain human behavior independently of culture-specific features and to find general, universal traits (for a more detailed explanation of the terms see Morris et al., 1999). Even though human nature has some universal characteristics, there are underlying culture-relative assumptions that make the etic approach inappropriate for psychiatry.

The various normative elements implicit in the assessment of psychiatric disorder overlap, and much of what has been said above about the concept of harm, moral frameworks, and even the question of rationality could have its place in this section as well. Therefore, what I am going to do in this section is only to highlight the various cultural dimensions of psychiatry. These are assumptions and mechanisms regarding the *causes* of mental disorder, the impact of culture on *diagnosis*, specific differences in the individual *experience* of mental disorder, and last but not least the *evaluation* of behavior from the third-person perspective.

#### Causes

Culture or the character of a given society seems to influence the development and understanding of psychic problems both directly and indirectly; indirectly through the norms and social expectations the individual has to follow, directly through the expected ways of behavior which determine deviance. In an interesting article Catherine Caldwell-Harris and Ayse Ayçiçegi formulated a “*personality-cultural clash hypothesis*” according to which there is a correlation between personality-style, cultural character and mental health (Caldwell-Harris and Ayçiçegi, 2006). They state that “[p]ersonality traits associated with psychopathology will be most frequent in allocentrics living in an individualist society, and in idiocentrics living in a collectivist society.” In collectivist societies where strict rules of social behavior have to be followed and social harmony is highly valued, people with an idiocentric (extremely individualistic) personality tend to have poorer mental health with high scores in paranoid, schizoid, narcissistic, borderline, and antisocial PDs. In individualistic societies, by contrast, a distinct allocentric (extremely collectivist) personality is positively correlated with social anxiety, depression, obsessive-compulsive disorder, and dependent personality. In addition to this indirect influence on mental disorder, there is a more direct influence, too. This can best be illustrated by Wakefield's account of cultural relativity:

“Whereas social phobia is a real disorder in which people can sometimes not engage in the most routine social interaction, current criteria allow diagnosis when someone is, say, intensely anxious about public speaking in front of strangers. […] This diagnosis seems potentially an expression of American society's high need for people who can engage in occupations that require communicating to large groups.”

(Wakefield, 2007, p. 154)

In sum, not only has the respective cultural setup an indirect influence on mental health, it also tends to dictate the boundary between the normal and the deviant on the basis of the expected values and virtues of its members. In this respect the impact of society on the concept of mental disorder is clearly normative. Whether the indirect influence, i.e., the personality-cultural clash, turns out to be directly normative under the surface after all remains an issue for further scrutiny.

#### Diagnosis

Culturally specific views on psychiatric problems are harder to detect in our era of mass migration and globalization than in earlier times with more stable national and cultural boundaries. Nonetheless, important cultural differences regarding mental disorders remain, to which I am only able to allude in the following. What is more, the culturally formed experiences of psychic problems are not only to be considered on the patient's side but also on that of the practitioner, as Laurence Kirmayer points out (Kirmayer, 2001). This has also been shown some years ago by a study that compared the diagnostic patterns of American and Japanese clinicians (Tseng et al., 1992).

Three points regarding psychiatric diagnoses should be stressed here. Firstly, many mental disorders indeed really “exist” in the sense that they are modes of experiencing oneself and the world which are extraordinarily burdensome. Secondly, experience and behavior can only be understood against the background of other people's behavior and experience. Social phobia, for instance, presupposes a social surrounding not only because it is the very object of the phobia but also because it constitutes the basis of comparison against which a person assesses her own experiences. Thirdly, since there are “real” disorders on the one hand and dynamic social expectations on the other, it follows that the boundary between average and deviant behavior cannot be but normative. This is *not* just due to epistemological limits. Those boundaries simply do *not exist* by nature. What should psychiatrists do who are in need of a boundary that does not exist? They have to define it themselves (with the help of their social community) and put up a sign that reads “Attention, you are leaving the normal sector!” Seen in this light it is hardly surprising that there appears to be an extreme variance of prevalence rates for, e.g., social anxiety disorder across cultures, ranging from 0.2% in China and 7.9% in the US to 44.2% in rural areas of Udmurtia, a Constituent Republic of the Russian Federation (Hofmann et al., 2010, p. 118). Even if this spectrum should be primarily due to differences in case finding methods and there is in actual fact no “real difference in major psychiatric disorders across cultures and societies” as Andrew Cheng assumes (Cheng, 2001), it nevertheless mirrors all the problems and dependencies of psychiatric diagnosis and, hence, the impact of cultural and other norms and values on it.

#### Experience

Are psychological problems all the same around the world? If they are, science may be in a position to explain them on a purely molecular level one day. Two very common examples shall suffice at this point for an illustration that this is a vain hope. First, it is well known—even though hotly debated—that depression in Asian societies is experienced more as bodily malaise by the persons affected. The western counterpart of this “somatization” is sometimes called a “psychologization” (cf. Kirmayer, 2001). The Vietnamese language, for example, does not even have words for psychiatry, schizophrenia, and depression (Phan and Silove, 1997). A similar striking cultural difference can be found in the case of social anxiety. While in the western cultural sphere this is connected with the fear of being harmed or offended, in Japan and Korea people are in fear of harming or offending *others* (taijin kyofusho). Admittedly, taijin kyofusho is—along with other culture-specific disorders—at least mentioned in the DSM as well as in the ICD, but whether it is the same social anxiety disorder as in the western world, maybe a cultural-specific expression of it, or a disorder in its own right, is still under debate (cf. Hofmann et al., 2010). If two psychological problems that are quite differently experienced by the patients in different cultures get explained with one and the same molecular configuration, does this not come down to a Procrustean bed into which diagnoses are forced? Both expressions of social anxiety arise from and are judged by social norms.

#### Evaluation

As repeatedly mentioned in this article, whether a certain kind of behavior or experience counts as deviant and (potentially) as a psychological problem is often (even though not always) due to specific socio-cultural expectations. Somebody who is “dynamic” in one cultural region may be regarded as offensive in another. Remember the abovementioned western girl in rural Turkey (or the other way round). Here, expectations of rationality, morality, harm and harming combine to a normative framework against the background of which behavior is assessed and disorders are diagnosed. That does not mean there are no culturally and normatively independent mental disorders at all. But it would nevertheless be a fallacy to deduce the thesis that norms do not play a significant role in the assessment of mental disorder from their undisputed existence.

### Routes of explanation

Three levels of observation are of particular relevance in psychiatry. These levels exist in other areas as well, but when it comes to mental health and the concept of mental disorder, they have particularly far-reaching implications. These are the *explanatory level*, the *phenomenal level*, and the *interventional level*. One might use “reflection” instead of “observation,” but since “reflection” is in some sense too ambitious a word, associated with deep scrutiny and deliberation, “observation” is more adequate, as will become clear in the following.

Let's begin with the *explanatory level*. Here we find all the traditional models of explanation such as *the psychoanalytical* (Freud), the *sane reaction* model (Laing), the *labeling* model (Rosenhan), the *problems of living* account (Szasz), the biopsychosocial model (Engel), or the currently dominating *medical* model. It will make a considerable difference if you claim with Szasz that mental diseases just do not exist, assume with Rosenhan that it is largely a matter of labels, or if you search for purely biological causes. Each of these models of mental disorder constitutes a basic explanatory *norm* since there just is no higher level of objectivity from which we could assess the validity of one explanatory account or the other. Admittedly, we can (and do) use the effectiveness of an explanation and its respective therapies as a criterion, but whether psychopharmacological means are the most effective ones is open to debate even today. Hence, everything depends on questions of the philosophy of science, ontology, causality and—on an even deeper level—on the question of what constitutes an explanation.

On the *phenomenal level*, what kind of behavior or experience indicates a mental disorder depends on all the factors discussed above. The phenomenal level is in itself independent of a particular mode of (causal) explanation. Often it is just a matter of tradition or even intuition. The important aspect is that pathologic behavioral deviance is assessed through its “being different.”

On the *interventional level* mental disorders are seen in the perspective of therapy, i.e., a successful cure is already part of the explanation of a particular disease.

The *routes of explanation* come into play when we ask where to start in order to understand the nature of mental disorders. It is an interesting phenomenon that we may come to quite different results, depending on where we start. If we begin at the explanatory level, psychiatric disorders may disappear if we are followers of Szasz, or turn out to be purely physical if we adhere to the medical model. In the first case mental disorders cease to be, in the second they cease to be mental. In the first case we do not need a therapy, in the latter the therapy will probably be a pharmacological one. We will get similar “start-dependent” results with the psychoanalytical or the biopsychosocial model. What is important here is that what we assume on the explanatory level defines what we believe on the other levels.

The same holds true for the other routes. If we start on the level of interventions and make use of pharmacological therapies, we will probably come to the conclusion that psychiatric disorders are indeed something physical. In this case we are even in danger of getting ourselves into a circle: Why are pharmacological therapies indicated? Because psychiatric disorders are brain defects. How can we know that psychiatric disorders are brain defects? We can conclude this from the effects of our pharmacological therapies (cf. Valenstein, 1998, p. 222). To give a third and last example: If we believe some behavior to be strange and pathologic, we will surely find a cause of it at the explanatory level. So we have come full circle: Remember the quote from Szazs at the beginning, that “being kind to one's wife is not the sort of behavior to which we want to assign a causal (psychiatric) explanation.”

## Epilogue

The fact that our understanding of mental disorders is guided by several kinds of norms does not mean that these disorders do not exist. More precisely, on the one hand there is psychological suffering which can hardly be doubted in its existence, relevance, and “realness.” On the other hand there are several cases of mental “disorder” which clearly rest on direct and indirect, open and covert normative assumptions. This has at least two consequences. First, psychiatric disorders are not “out there” and not to be understood as objectively discoverable entities that can always be separated from each other. The boundaries between normal and non-normal behavior and those between one disease category and the other are floating. Second, because of the normative nature of psychiatry, mental disorders cannot be completely reduced to neuronal or molecular processes. Again, more precisely: A mental state as such may well be reducible to the brain, but determining whether this very mental state is (part of) a *disorder* or not is nothing the brain sciences can do. Something will always be lost in translation.

### Conflict of interest statement

The author declares that the research was conducted in the absence of any commercial or financial relationships that could be construed as a potential conflict of interest.

## Footnotes

^1^In the following I will use “mental,” “psychiatric,” and “psychological” disorder interchangeably. Likewise the term “behavior” is used as a placeholder that stands for “experience, emotion, and behavior.”

^2^Imagine a neurologist tapping with her finger on your brain scan and telling you “Oh, look, you were quite depressed last week.”

^3^Paquette and colleagues put it the other way round: “Change the mind and you change the brain” (Paquette et al., 2003). This is, of course, true as well, but not because of some spooky sort of mental causation, but rather because changing the mind *just means* changing the brain.

^4^The original DSM-IV criteria are: 301.38 (1), “frantic efforts to avoid real or imagined abandonment”; 301.6 (7), “urgently seeks another relationship as a source of care and support when a close relationship ends”; 301.6 (8), “is unrealistic preoccupied with fears of being left to take care of himself or herself.”

^5^The following four frames of reference are oriented toward those of Leising et al. (2009) but are not completely identical to them.

^6^This holds notwithstanding the assumption of a set of ubiquitous virtues (courage, justice, humanity, temperance, wisdom, and transcendence) shared in all cultures (Dahlsgaard et al., 2005).

^7^Bunge distinguishes seven concepts: conceptual, logical, methodological, epistemological, ontological, and valuational rationality (Bunge, 2007, p. 117 f.).

^8^I am only mentioning Wakefield's concept of “dysfunction” here without having room for a discussion.
